# Cystic hygroma and potential airway obstruction in a newborn: a case report and review of the literature

**DOI:** 10.1186/1757-1626-2-48

**Published:** 2009-01-13

**Authors:** Sulaiman Sannoh, Esperanza Quezada, David M Merer, Augustine Moscatello, Sergio G Golombek

**Affiliations:** 1 Division of Neonatology, Children's Regional Hospital, Cooper University Hospital, Camden, New Jersey, USA; 2 Division of Newborn Medicine, Maria Fareri Children's Hospital, Westchester Medical Center, New York Medical College, Valhalla, New York, USA; 3 Division of Otolaryngology, Department of Surgery, Westchester Medical Center, New York Medical College, Valhalla, New York, USA

## Abstract

**Background:**

Cervical cystic hygroma is a benign congenital malformation of the lymphatic system. Incidence of cystic hygroma is 1/6000 live births. We present a case of right neck mass with potential respiratory compromise in a newborn.

**Case presentation:**

The patient was a full term baby girl with an incidental finding of right neck mass which was described on ultrasound and magnetic resonance imaging as a cystic lesion in the nasopharynx and right neck which inferiorly followed the course of the right carotid artery, consistent with cystic hygroma. She started with respiratory compromise, and a follow-up magnetic resonance imaging showed increased size of the cystic hygroma. Dexamethasone was started to reduce fluid build up in the mass. When the cystic hygroma was found to be inseparable from the right half of the thyroid gland, the otolaryngologist performed hemithyroidectomy.

**Conclusion:**

The patient had neuropraxia involving the marginal mandibular branch of the facial nerve, which was expected to correct with time. Large cervical cystic hygromas may surround or displace neurovascular structures making their identification quite challenging intraoperatively. A team of experienced surgeons will help to ensure a successful surgical outcome.

## Background

Cystic hygroma is a benign congenital malformation of the lymphatic system that has its genesis in the lack of development of communication between the lymphatic and venous systems. The cyst may be unilocular or multilocular and could be of variable size but is characteristically brilliantly transilluminant. The incidence of cystic hygroma is approximately 1/6000 live births [1]. 70–80% of cystic hygromas occur in the neck, usually in the posterior cervical triangle [2]. The remainder 20–30% occurs in the axilla, superior mediastinum, chest wall, mesentery, retro-peritoneal region, pelvis and lower limbs [3]. Cystic hygroma is known to present at birth in about 50% of the affected newborns and 90% present by age 2 years [4].

We present the case of a right neck mass which was initially asymptomatic but eventually caused stridor, and vomiting episodes after feeding

## Case report

A 3.3 kg full term African American female infant was delivered at a community hospital by cesarean section secondary to fetal bradycardia and suspected macrosomia to 37 years old G8P5 mother. The pregnancy was complicated by gestational diabetes mellitus and pregnancy-induced hypertension. Apgars were 8 and 9 at 1 and 5 minutes respectively. No neck mass was reported on prenatal ultrasound. The lack of maternal fetal medicine specialists at the community hospital might explain the quality of prenatal ultrasound. In the well baby nursery at the community hospital a right neck mass was noted. Due to history of spitting on regular infant formula, soy-based formula was started, but without improvement. She was transferred to the Regional Neonatal Center for further evaluation and management of the neck mass.

**Figure 1:**
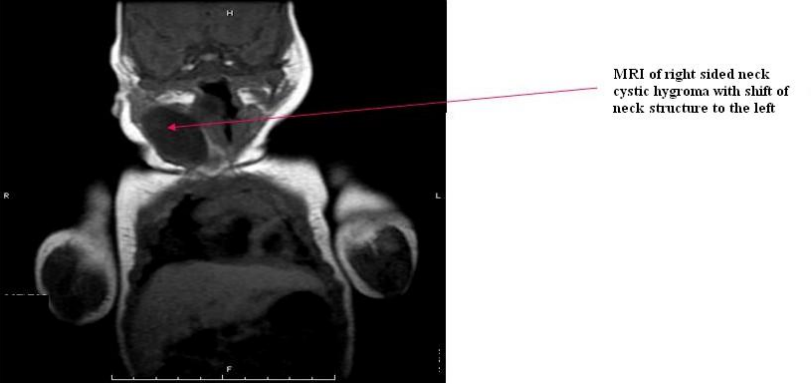
**Initial magnetic resonance imaging of the neck showing right sided cystic hygroma, pushing the neck structures to the left**.

**Figure 2:**
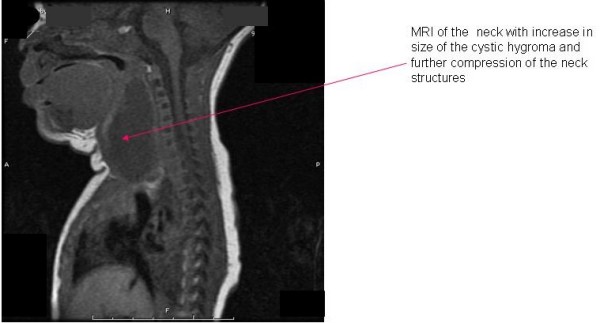
**Follow-up magnetic resonance imaging of neck showing increase in size of the cystic hygroma with further compression of the structures of the neck**.

The baby was on room air for 1 week when she developed stridor. The neck mass had increased in size and a Pediatric ENT consult was obtained. An MRI demonstrated "a cystic lesion in the nasopharynx and right neck which inferiorly followed the course of the right carotid artery probably consistent with cystic hygroma" [fig. 1]. The mass continued to increase in size so a follow-up MRI and MRA were done. MRI showed increase in size of right posterior retropharyngeal to right neck cystic mass measuring 4.1 cm and displacing the trachea and oropharynx to the left and the right carotid artery posteriorly and laterally [fig. 2]. MRA showed the right common carotid artery displaced posteriorly and laterally without definite hemodynamic stenosis. But it is possible that neurovascular and respiratory structures of the neck were compressed during labor to cause fetal bradycardia. Carotid duplex ultrasound was recommended, which was severely limited due to displacement of vessels, presence of the mass and small size of the neck. To reduce fluid build up in the neck mass, the patient was started on 0.1 mg/kg of dexamethasone every 6 hours after the first MRI. She was later intubated for stridor and concern of potential airway obstruction. A team of pediatric otolaryngologists and pediatric surgeons was assembled to address the mass. The otolaryngologist decided to approach the mass via a cervical incision. Imaging studies demonstrated that the mass extended inferiorly to the aortic arch and a sternostomy had been planned. Intraoperatively the mass was found to be limited to the neck, so a sternostomy was not necessary. A hemithyroidectomy was required when the cystic hygroma was found to be inseparable from the right half of the thyroid. Dexamethasone was tapered over 2 weeks. She was observed for 24 hrs after the steroid taper. Endocrinology was consulted after the hemithyroidectomy. Initial thyroid function tests were normal except for mildly elevated T4 [13.9 μg/dL]. PTH was also normal [50.8 pg/L]. Genetics was consulted and chromosomal analysis showed 46 XX. The patient was noticed postoperatively to have a weakness of the right lower lip, consistent with neuropraxia involving the marginal mandibular branch of the facial nerve. This deficit is expected to correct with time.

## Discussion

We report an unusual case of neonatal neck mass. The mass was investigated with ultrasound, MRI/MRA and found to be a cystic hygroma of the neck and mediastinum. Cervical cystic hygromas or lymphangiomas are believed to occur as a result of the failure of establishment of appropriate connection to the normally present lymphatic channels. They are usually encountered at birth or in early infancy. Very few hygromas extend into the mediastinum [5]. Lymphangiomas may be divided histologically into two major groups based on the depth and the size of abnormal lymph vessels. The superficial ones are called lymphangioma circumscriptum. The more deep seated ones are cavernous lymphangioma or cystic hygroma [6]. Cystic hygromas are deeply seated in areas of areola or loose connective tissues. They appear early in life as large soft-tissue mass on the axilla, neck or groin. They are soft, vary in size and shape, and tend to grow extensively if not surgically excised. They are multilocular cysts filled with clear or yellow lymph fluid [7]. Usually cystic hygromas are diagnosed clinically with large size, location and translucence. Although cystic hygromas tend to enlarge progressively over months a relatively rapid increase in size has also been described [8]. Cystic hygromas may be associated with Turner syndrome, Noonan syndrome, trisomies, fetal alcohol syndrome, chromosomal aneuploidy, cardiac anomalies and fetal hydrops [9]. The management of lymphagiomas including cystic hygromas is preferably surgical, although a careful "wait and see" policy may be indicated in few asymptomatic cases, as spontaneous regression has been reported [10]. Indications for surgery in pediatric cases include significant cosmetic deformity, obstructive symptoms, bleeding and recurrent infections. Other treatment modalities include aspiration, radiation, and injection of sclerosing agents, in particular the agent OK-432, derived from a strain of streptococcus pyogenes, which has been used successfully, especially in macrocystic lymphangiomas and in patients who are at increased anesthetic risk [11]. It is not uncommon for infants to develop neural paresis or paraylsis after excision of massive cervical lymphangiomas. These congenital abnormalities tend to distort normal anatomy. They may surround or displace neurovascular structures making their identification quite challenging intraoperatively. A team of experienced surgeons from varying fields including Otolaryngology, Cardiothoracic and Pediatric Surgery will help to ensure a successful surgical outcome. Postoperatively, Endocrinology may need to be involved to monitor surgery-related endocrine dysfunctions.

## Conclusion

Cervical cystic hygroma with mediastinal extension can lead to respiratory and neurovascular compromise.

## Abbreviations

MRI: magnetic resonance imaging; MRA: magnetic resonance angiography; PTH: parathyroid hormone; ENT: ear, nose and throat surgeon.

## Consent

Written informed consent was obtained from the mother of patient for publication of this case report and accompanying images. A copy of the written consent is available for review by the Editor-in-Chief of this journal

## Competing interests

The authors declare that they have no competing interests.

## Authors' contributions

All authors contributed to acquisition of case details and the analysis and interpretation of them. SS wrote the first draft of the manuscript, SGG, DMM, AM, EQ revised the manuscript. All authors have given final approval of this version to be published
